# Radio Frequency Resonator-Based Flexible Wireless Pressure Sensor with MWCNT-PDMS Bilayer Microstructure

**DOI:** 10.3390/mi13030404

**Published:** 2022-03-01

**Authors:** Baochun Xu, Mingyue Li, Min Li, Haoyu Fang, Yu Wang, Xun Sun, Qiuquan Guo, Zhuopeng Wang, Yijian Liu, Da Chen

**Affiliations:** 1 College of Electronic and Information Engineering, Shandong University of Science and Technology, Qingdao 266590, China; xbcno12022@163.com (B.X.); lxm_0529163@163.com (M.L.); minlee163@163.com (M.L.); fanghy0820@163.com (H.F.); wangyunn1119@163.com (Y.W.); wzhuopeng@126.com (Z.W.); 2 Guizhou Aerospace Institute of Measuring and Testing Technology, Guiyang 550009, China; sunxunup@alumni.sjtu.edu.cn; 3 Shenzhen Institute for Advanced Study, University of Electronics Science and Technology of China, Shenzhen 518110, China; guoqiuquan@uestc.edu.cn

**Keywords:** wireless, passive, radio frequency, pressure sensor, non-contact sensing

## Abstract

Flexible pressure sensors have been widely applied in wearable devices, e-skin, and the new generation of robots. However, most of the current sensors use connecting wires for energy supply and signal transmission, which presents an obstacle for application scenarios requiring long endurance and large movement, especially. Flexible sensors combined with wireless technology is a promising research field for realizing efficient state sensing in an active state. Here, we designed and fabricated a soft wireless passive pressure sensor, with a fully flexible Ecoflex substrate and a multi-walled carbon nanotube/polydimethylsiloxane (MWCNT/PDMS) bilayer pyramid dielectric structure. Based on the principle of the radio-frequency resonator, the device achieved pressure sensing with a changeable capacitance. Subsequently, the effect of the pyramid density was simulated by the finite element method to improve the sensitivity. With one-step embossing and spin-coating methods, the fabricated sensor had an optimized sensitivity of 14.25 MHz/kPa in the low-pressure range. The sensor exhibited the potential for application in limb bending monitoring, thus demonstrating its value for long-term wireless clinical monitoring. Moreover, the radio frequency coupling field can be affected by approaching objects, which provides a possible route for realizing non-contact sensing in applications such as pre-collision warning.

## 1. Introduction

Flexible pressure sensors have been developing rapidly in many fields of application, including biomedical applications and wearable devices [1,2,3,4], and can efficiently realize the regulation of physiological state, allowing timely intervention for the protection of human health, and resulting in great improvement to the quality of human life [5,6,7,8]. However, most present flexible pressure sensors require redundant wires and batteries, which presents a considerable challenge in miniaturized scenarios, as well as those requiring movement [9,10], such as implant devices, limb motion [8,11,12], rotating environments [13,14], and the inside of pipes [15,16]. Meanwhile, systems that do not require large power sources make sensors more convenient for continuous detection and processes necessitating a longer endurance. Based on the radio frequency principle, passive radio-frequency sensors (RF sensors) provide a reasonable solution for the realization of miniaturized, radio-powered, and long-endurance systems [17,18].

RF sensors can be considered as an inductance-capacitance (LC) resonant tank that is coupled with and powered by a radio frequency readout antenna. The LC resonant tank is typically constructed with a spiral inductance connected to a sensing capacitor. The L (inductance), C (capacitance), and R (resistance) change relatively in response to the deformation of the sensor, resulting in a shift in its resonant frequency. According to current research in LC-based sensors, capacitive sensing is the most reasonable method, where the parameter of interest is plate distance [19].

The sensitivity and detection range are the most important indicators for evaluating the measurement accuracy and capacity of pressure sensors. High sensitivity leads to high detectable pressure resolution, allowing applications for the detection of small stimuli in sensing systems. Several recent studies have shown that microstructures, including spongy structures [20], intrafillable microstructures [1], and micro-pyramid arrays [21], are effective solutions for improving sensitivity. In addition, carbon nanotubes [22], graphene [23], nanowires [24], and their doped composites have emerged as a hotspot in attempts to achieved improved sensitivity. This improvement is somehow due to the numerous parallel capacitors formed by the conductive nano-particles, which causes an increase in the dielectric constant [25,26]. However, excessive sensitivity usually confines the detection range, since, because of the limited deformation space of the sensitive part, a trade-off must be made between sensitivity and detection range. More importantly, methods for introducing microstructure and doping materials into the LC structure in order to improve sensing performance is of great significance in applications such as wearable electronics, e-skin, and soft robots.

Herein, we report a highly sensitive pressure sensor based on a flexible radio frequency resonator characterized by a multiwalled carbon nanotube (MWCNT)-doped Polydimethylsiloxane (PDMS) dielectric medium with a high dielectric constant and a bilayer pyramid structure for the detection of tiny forces applied on the device surface. Based on the principle of the parallel plate capacitor, the bilayer pyramid dielectric formed by the MWCNT-doped PDMS achieves a high sensitivity and a wide operation range, while also demonstrating good signal quality. We introduce a one-step embossing method to fabricate the conductive circuit on Ecoflex substrate. The functional circuit contains capacitance plates, an inductance coil, and conductive wire. Furthermore, the resonant tank consists of an overturned sandwich structure in order to perform pressure sensing. A finite element analysis (FEA) simulation was built to optimize the structure. To demonstrate the feasibility of its application, the sensor was used to detect pressure changes while a limb was performing activities and during subtle physical motions. In addition, based on the electromagnetic coupling principle, the RF sensor is also able to perform non-contact sensing. With these excellent properties, it is believed that our RF pressure sensor possesses extensive application prospects in human movement monitoring, soft robot control, and medical treatment.

## 2. Material and Methods

### 2.1. Sensitive Mechanism

**Figure 1:**
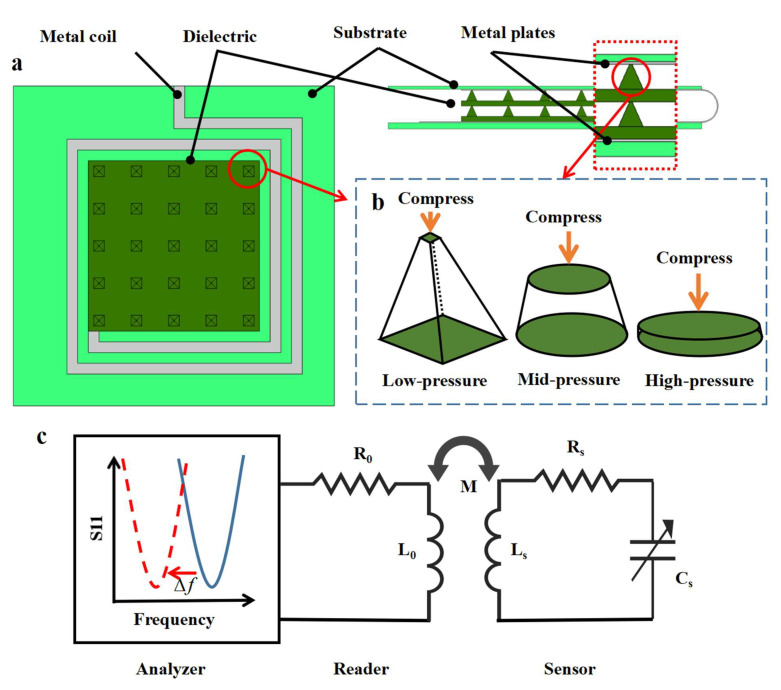
**Schematic illustration of mechanisms and sensing of the radio-frequency (RF) pressure sensor.** (**a**) Top view and side view of the RF sensor structure with the marks of main components. (**b**) The equivalent geometric changing model of the pyramid structure while under stress. (**c**) Diagram of the S11 curve changing while under stress and schematic circuit model of the RF sensor.

The device was formed by folding the Ecoflex substrate with a metal circuit on it, sandwiching the MWCNT/PDMS bilayer dielectric structure (Figure 1a). The metal circuit is regarded as a resonant tank constructed by a two-turn spiral inductance connected with a plane-parallel sensing capacitor.

As shown in Figure 1c there is electromagnetic coupling between the readout antenna and the inductance of the sensor, which changes the distance between the capacitor plates, resulting in variation in the reflection loss of the readout device. This means that the status parameters of the sensor can be obtained by continuously sending a sine frequency sweep signal within a certain frequency range to the one-port network [19]. In principle, the thickness of the entire dielectric layer will be changed by the pressure exerted on the sensor, thus providing variation in the capacitance of the circuit and causing a frequency shift. Pressure sensitivity (*S*) is defined as the absolute decrease of the resonant frequency ($\Delta \;f_{s}=f-f_{0}$) per unit pressure (ΔP).

(1) S=ΔfsΔP=−1ΔP(14π(LsCs)32)(CsΔLs+LsΔCs)=−kΔCsΔP,

(2) k=Ls4π(LsCs)32,

where $\Delta L_{s}=0$, $\Delta C_{s}\propto \Delta h$, Δh is the height of the deformation, and *L_s_, C_s_* are initial constants of the circuit. Therefore, the greater the deformation under a certain pressure, the higher the sensitivity possessed by the sensor. In this work, multi-walled carbon nanotubes (MWCNT) were doped into the PDMS as a composite material to increase the permittivity.

The elastomeric dielectric layer can be modeled as springs [27]. The equivalent spring constant k is described and combined with Hook’s law as

(3) $$\Delta h=\frac{F}{k}=\frac{\Delta Ph_{0}}{E_{e}},$$

Substituting Equation (3) into Equation (1) gives

(4) $$S\propto \frac{1}{E_{e}},$$

The inverse relationship between the equivalent modulus *E_e_* and sensitivity *S* indicates feasible guidance for the design of sensor structures. In Figure 1b, a single micro-pyramid is geometrically presented under low pressure, and the equivalent modulus of this layer (*E_e_*) can be estimated as

(5) $$E_{e}=E\frac{V_{e}}{V_{s}}=E\frac{n}{V_{s}h_{0}}\int_{0}^{h_{0}}{\left(\frac{h_{0}-h}{h_{0}}\right)}^{2}dh,$$

where *h*_0_ is the initial height of the pyramid, E is the young’s modulus, and *V_e_*, *V_s_* are the effective volume and total sensing volume. Because the volume of the micro-pyramid is smaller than that of the flat structure, a low modulus, and thus a high sensitivity, can be obtained. The high Poisson’s ratio (0.49) of PDMS results in vertical expansion converging with horizontal expansion. Due to the isotropy of the expansion process, the pyramid expands in a circular plane under the expansion conditions. Meanwhile, under mid-pressure, the pyramid forms into a cylinder, and thus the equivalent modulus (*E_e_*) can be expressed as

(6) $$E_{e}=E\frac{V_{e}}{V_{s}}=E_{e}\frac{n}{V_{s}h_{t}}\int_{0}^{h_{t}}\frac{\pi }{4}dh,$$

where *h_t_* is the compressed height of the pyramid. It can be seen that the equivalent modulus increases with increasing pressure. When the force reaches the high-pressure range, the pyramids are entirely compressed, and so that the dielectric structure can be regarded as a flat elastomer. In this case, the equivalent modulus (*E_e_*) is about the same as the Young’s modulus E. The deformation conditions are defined in accordance with the three phases of the test results as mid-pressure (4–20 kPa) and high-pressure (20–60 kPa). In particular, the separation distance of the pyramid array had a great impact on the resistant modulus of the total structure, owing to the relative volume of the elastomer. Additionally, the bottom pyramids support the flexible membrane structure of the upper layer. Due to the low modulus of the PDMS, there are waves in the upper membrane waves at the same time as the compression of the pyramids is occurring, making a joint contribution to the total structural strain. This pyramid-membrane co-bearing effect means that the use of the bilayer structure does not simply involve spatial stacking, but also amplifies the compression deformation and thus amplifies the sensitivity. To optimize the performance of the sensor, including the sensitivity, working range, repeatability, and frequency stability, several pyramid arrangements with different separation distances were compared.

### 2.2. Structure Optimization

**Figure 2:**
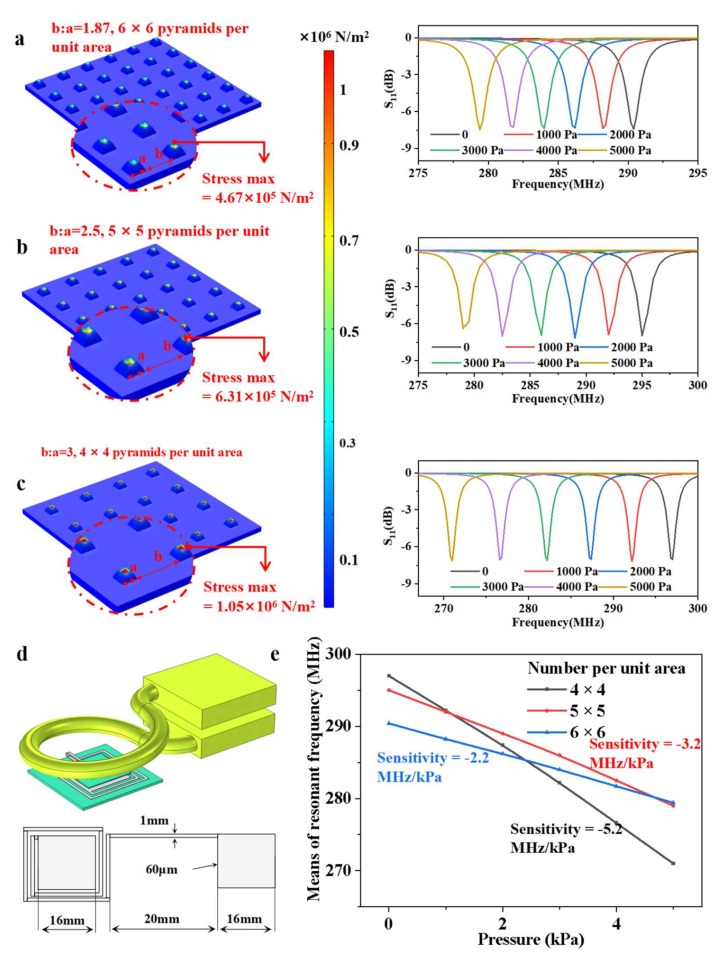
**Optimization of the pyramid array structure using finite element analysis software.** Schematic diagram of the dielectric layer with various pyramid arrays, including 6 × 6 (**a**), 5 × 5 (**b**), 4 × 4 (**c**). (**a**) Stress distribution and S11 curves of the sensor with a 6 × 6 pyramid array versus pressure from 0 to 5 kPa in steps of 1 kPa. (**b**) Stress distribution and S11 curves of the sensor with a 5 × 5 pyramid array versus pressure from 0 to 5 kPa in steps of 1 kPa. (**c**) Stress distribution and S11 curves of the sensor with a 4 × 4 pyramid array versus pressure from 0 to 5 kPa in steps of 1 kPa. (**d**). Geometric schematic with specific parameters and the electrically coupled module built by COMSOL simulator. (**e**) Sensitivity curves of the sensor with three densities of pyramids.

The simulation model was performed using the COMSOL Multi-physics simulator (v5.5, COMSOL inc., Stockholm, Sweden). Figure 2 shows the simulation results for a sensor consisting of a single layer with a certain pyramid size and various separation distances. The typical material parameters of PDMS and Ecoflex are given in Table A1 and Table A2, with the aim of providing qualitative design guidance. Figure 2a–c show the stress distribution schematics under 1000 Pa pressure, and the S11 response curves of a sensor with a single-layer pyramid array. The geometric schematic with specific parameters and the electrically coupled module built using the COMSOL simulator are shown in Figure 2d. As summarized in Figure 2e, the simulated resonant frequency was a nearly linear function with increasing pressure over a range of 0–5 kPa. The sensitivity of a single-layer sensor with 6 × 6 pyramids per unit area was about −2.2 MHz/kPa. The changes in resonant frequency changes of a sensor with a 5 × 5 array were improved to −3.2 MHz/kPa, and with a 4 × 4 array, this value reached −5.2 MHz/kPa. Due to the differences in dielectric composition and the proportion of PDMS to air, different sensors have slightly different initial frequencies. The simulation results illustrate that the higher the separation distance is, the higher the sensitivity will be. However, considering the dead load of the sensor itself, the separation distance is limited to below a critical value. This threshold can be flexibly changed, depending on various application needs. The experimental data and simulation data are summarized in Table A3.

### 2.3. Device Fabrication

**Figure 3:**
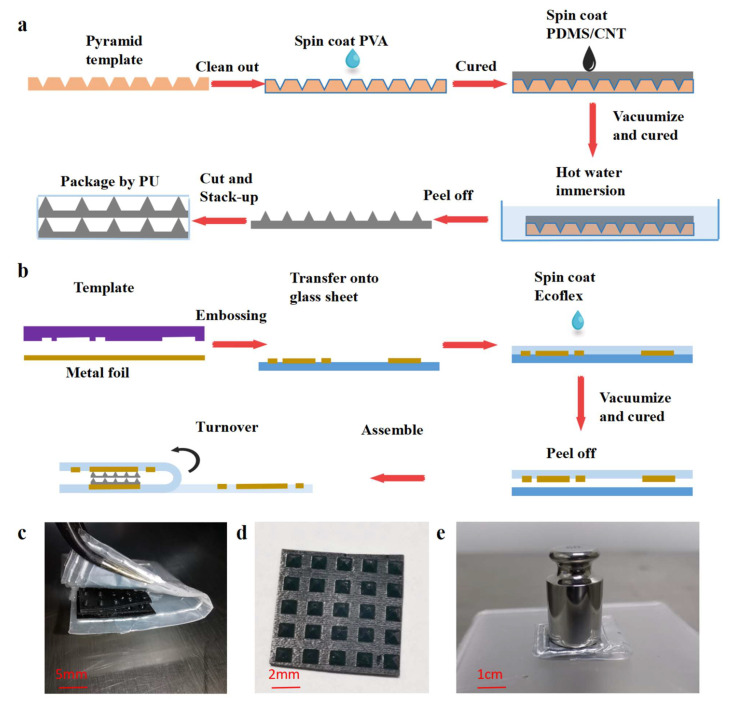
**Schematic of the fabrication process and photos of** the **finished devices.** (**a**) Process for fabricating the bilayer pyramid dielectric with a template. (**b**) Process for manufacturing the metal–Ecoflex film and assembly of the sensor. (**c**) Overview of the sensor showing the detail of the dielectric structure. (**d**) Top view of the fabricated pyramid array. (**e**) Photo of the device under pressure.

The manufacturing process for the RF pressure sensor is shown in Figure 3a,b. The three main steps were the fabrication of the bilayer dielectric structure (Figure 3a), the metal pattern and adhesive with the Ecoflex substrate, and their assembly into a device (Figure 3b).

The bilayer pyramid array dielectric structure was formed on a PDMS film using a hollowed-out model as the template. In the first step, polyvinyl alcohol solution (PVA, 50 mg/mL, from Shandong Yousuo Chemical Technology Co., Ltd., Heze, China) was spin-coated (300 rad/s, 30 s) onto the surface of the pre-cleaned hollowed-out model and cured at 80 °C for 5 min. In the second step, MWCNT/PDMS mixture was spin-coated onto the template and placed in a vacuum oven (80 °C) for 30 min. Before that, the MWCNT (XFM06, Nanjing XFNANO Materials Tech Co., Ltd., Nanjing, China, < 8 nm diameter, > 95% purity) and polydimethylsiloxane (PDMS curing agent ratio, 1:15, Syl Gard 184 Silicone Elastomer Kit, Dow Corning, Shanghai, China) were mixed thoroughly by stirring and sonication. After that, the template coated with MWCNT/PDMS composite layers was dipped into hot water (50 °C) under an ultrasonic bath for 2 h to remove the sacrificial PVA layer. The pyramid dielectric layer was peeled away from the template before being cut to the appropriate size and heaped up as a bilayer. In addition, the bilayer pyramid array structure was kept in place by polyurethane (PU) bondage.

Figure 3b depicts the preparation of the metal–Ecoflex adhesive film and the assembly process. A layer of PVA was spin-coated onto a cleaned glass sheet for preparation. The metal foil was patterned using an embossing method, making it possible to form it into any shape, following the template. As can be seen in Figure S1, the hard template was put together with the copper conductive tape, and they were placed between two pressure plates. Then, the plates were passed through the embossing channel. After embossing, the excess part of the composite layer was separated from the template with tweezers to obtain a metal pattern with the desired structure. After the embossing process, the metal circuit was conformally transferred into the glass sheet that was coated with PVA. The last step was to uniformly spin-coat the vacuum-treated Ecoflex mixture (Smooth-on 0030, A: B ratio 1:1, 50 rad/s, 30 s. (Smooth-on Inc., Macungie, PA, USA)) onto the top surface, followed by curing in a vacuum chamber at 100 °C for 30 min to build stable self-crosslinking between the metal and the Ecoflex substrate. By using the same method for dissolving the sacrificial layer, the metal–Ecoflex combined substrate was easily peeled off.

Finally, the dielectric layer was placed upon one electrode plate, and then the other plate was turned over in alignment with the former. After fixing the construction using adhesive solution (Kisling, Bern, Switzerland), a stable RF pressure sensor was made. The fabricated sensor and details of the pyramid array are shown in Figure 3c,d, respectively.

## 3. Experiment Results and Discussion

### 3.1. Sensing Characteristics

**Figure 4:**
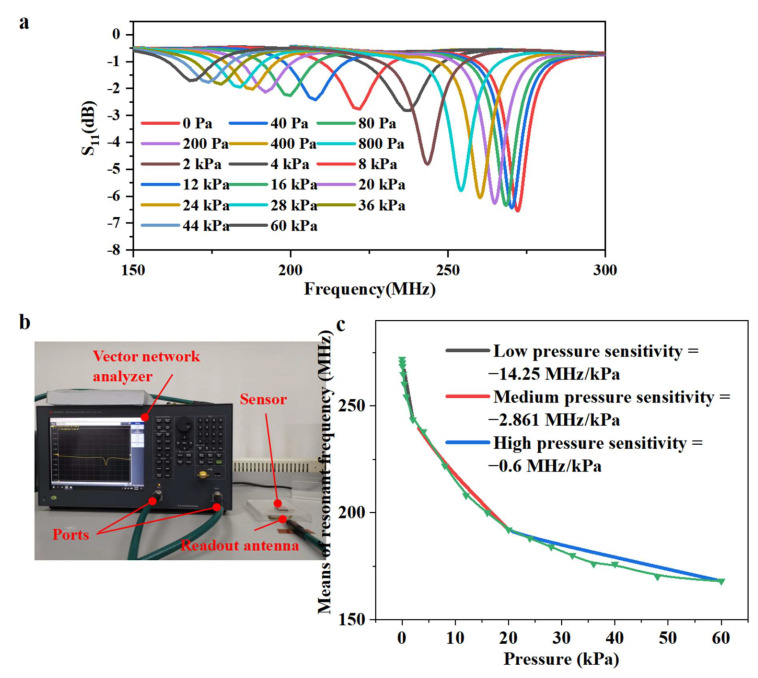
**Response characteristics of RF pressure sensors.** (**a**) The S11 responses of the sensor with bilayer pyramids structure under pressure gradually increase during low-pressure (0–4 kPa), mid-pressure (4–20 kPa), and high pressure (20–60 kPa) range. (**b**) Optical photograph of the test platform. (**c**) Sensitivity curve of the sensor with multi-walled carbon nanotube/polydimethylsiloxane (MWCNT/PDMS) bilayer pyramid array dielectric structure.

Figure 4 shows the collected resonant peaks curve and typical S11 response curves, respectively. Considering the dead load of the functional structure itself, a medium separation ratio (b: a = 2.5, 5 × 5) was chosen for testing sensor performance. The sensor showed three sensitive behaviors in the low-pressure range (0–4 kPa), mid-pressure range (4–20 kPa), and high-pressure range (20–60 kPa). The resonant frequency of the sensor decreased from 272 MHz (0 kPa) to 238 MHz (4 kPa), 192 MHz (20 kPa), and 168 MHz (60 kPa). These results also validate our theoretical analysis. When the pressure was below 4 kPa, the sensitivity of the sensor reached −14.25 MHz/kPa. Even when the applied pressure increased into the high-pressure range, the sensitivity of the sensor (−0.6 MHz/kPa) was still higher than the maximum sensitivity achieved in the most recent studies [12,15,16,20,28,29,30]. In contrast, the sensor with a 5 × 5 bilayer pyramid dielectric structure possessed superior sensitivity as well as a considerable working range. Additionally, a further experiment was performed to verify the resilience of the sensor; as shown in Figure S2, the sensor responded consistently during the cycle of increasing and decreasing pressure.

### 3.2. Application in the Detection of Various Body Movements

**Figure 5:**
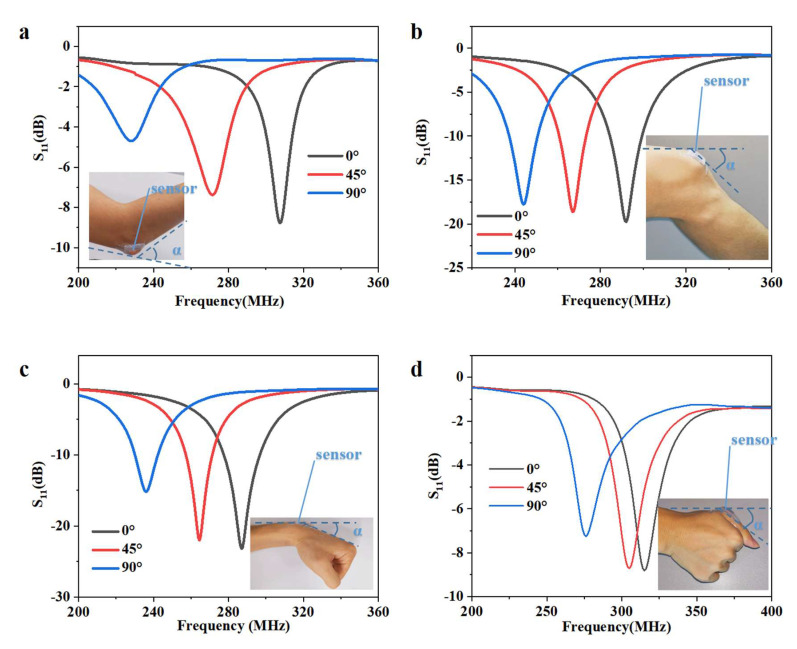
**Application in the detection of various human body movements.** Responses of the pressure sensor to (**a**) bending an elbow, (**b**) bending a knee, (**c**) bending a wrist, and (**d**) bending a finger.

As a result of their being fully flexible, the sensors are able to conform to human skin, remaining in contact and thus responding well to the body’s activities. Some typical applications for monitoring human motion and physiological activities are shown in Figure 5. To verify the practicality of the RF pressure sensor, the fabricated sensor was attached to the elbow, knee, wrist, and finger to measure the bending movements. Each resonant curve in the diagram corresponds to a specific bending angle. The initial frequency of the sensor exhibited large differences compared to both the static measurements and the measurements for various body positions. This is a result of the parasitic capacitance increase when the sensor is clinging to the human body, with the tightness of the bandage also influencing this. This problem could be resolved by correcting the baseline by recalculating the twisting stress according to the initial state after wearing the device. Notably, current popular data processing methods such as machine learning make this easy [31,32,33].

### 3.3. Non-Contact Sensing Ability

In addition, non-contact sensing has shown great potential in human–computer interaction, providing users with more freedom and a greater number of possibilities in practical devices. The ever-increasing application of smart electronics, including bio-friendly robotics, virtual reality, metagalaxy, and other supplementary devices, requires the use of bifunctional e-skin with both tactile and touchless properties [24,34,35,36,37]. One of the typical characteristics of RF sensors is that they can be affected by objects nearby without contact, thus making it possible to dissolve the now-common separation between tactile and touchless. This paradigm innovation possesses great prospects for removing the obstacles presented by redundant sensors within a VR system, enabling more complex interaction.

**Figure 6:**
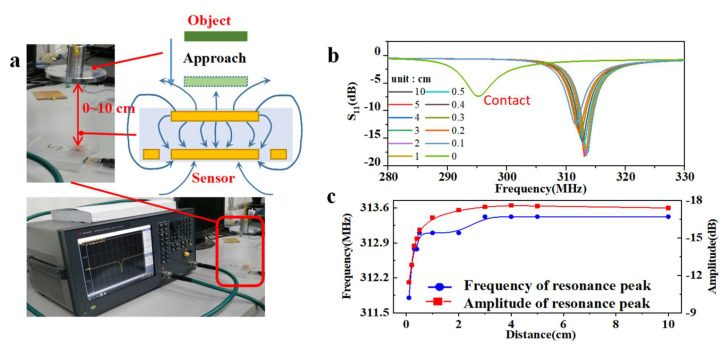
**The non-contact sensing ability of the RF sensor.** (**a**) Photos and schematic diagram of the non-contact sensing test. (**b**) Resonant frequency response curves at various distances. (**c**) Point curves of the frequency and amplitude of the S11 peaks.

Here, the RF sensor was subjected to a non-contact sensing test, as shown in Figure 6. It can be noticed from Figure 6c that the resonant frequency decreased gradually with increasing distance from 1 cm to 10 cm, while the decreasing tendency became steep at distances below 1 cm. Additionally, the amplitude of the curves decreased clearly to half at a distance of 0.1 cm compared to at a distance of 10 cm. During the object approach process shown in Figure 6b, the resonant frequency and amplitude decreased regularly with the approach distance, but the resonant frequency suddenly broke to 295 MHz at the moment of contact. This is an interesting phenomenon that has the potential to establish a unique contact-approach bifunctional sensing relationship using only one sensor. The reason for this break is hypothesized to be a change in stray capacitance or a somehow-introduced contact force. Specifically, as can be seen from the electric model presented in Figure 6a, the closer the object is, the more obvious the effect on the electric field is. According to the distribution principle of electric field intensity, the effect intensity will have a quadratic inverse relationship with distance. Therefore, at a very small distance before touch, the upper bound of the effect value will rapidly increase. The second notable factor is that the contact force cannot be completely avoided, and caused a response of about 15 MHz at a pressure of 1 kPa. Guided by this phenomenon, in future we will focus on developing the theoretical model and improving the experimental equipment in order to further refine the approach process and avoid various interference factors. This will make it possible to explore and validate the detailed scientific basis for this phenomenon, and to finally develop reasonable application scenarios.

The approach process can be regarded as being equivalent to a change in the capacitance of the environment around the sensor, which is also related to the polarity of the material and the size of the object. By comparing the three graphs in Figure S3, it can be seen that the effect of metal, body, and wood on the resonant frequency gradually weakened, but the difference was inconspicuous. This non-contact sensing combined with wireless interrogation allows the implementation of the concept of dual-terminal wireless sensing, which has great potential applications in fields such as VR-supplemented devices and the new generation of e-skin for soft robotics [35,38,39].

### 3.4. Scalability and Expectation for Arrays

Wireless pressure sensor arrays have many advantages for use in current hot topic areas such as electronic skin and human–computer interactions. In addition to the conventional advantages of wireless sensors, wireless sensor arrays also avoid the large amounts of wiring and pin arrangements, thus making it possible to read multiple cells in parallel. For example, as can be seen from the schematic shown in Figure S4, using micro-machining, multiple gradients of capacitors can be integrated, and the inductance coil can be shared so that multiple co-spectrum peaks of the array can be monitored using only one read antenna. By considering the frequency sweep, it is possible to obtain a huge amount of data, and the volume of multiple-unit information is large; therefore, some emerging data classification and prediction methods can be adapted, such as convolutional neural networks. Accordingly, wireless pressure sensor arrays using this sensor are expected to break through the current limitations of electronic skin with respect to spatial density and scale caused by wiring. At the same time, more technical approaches should also be explored, such as the improvement of reading concepts, and the convergence between emerging technologies and deep data mining.

## 4. Conclusions

In summary, we have reported a highly sensitive pressure sensor based on a flexible radio-frequency resonator. Based on the principle of the parallel plate capacitor, the variation in capacitance provides high sensitivity and a wide operation range, as well as stable signal quality. Benefitting from the bilayer pyramid dielectric structure and the high permittivity of MWCNT/PDMS, the optimized sensor demonstrates a sensitivity of −14.25 MHz/kPa in the low-pressure range (0–4 kPa). When the pyramids were compressed, the sensitivity dropped gradually, thus providing a wide working range with a uniform response until the pressure reached 60 kPa, which is a relatively large pressure under common working conditions. Additionally, several experiments were carried out to verify its feasibility in applications such as the monitoring of body movements. In addition, the approaching sensing test confirmed the potential value of dissolving the now-common separation between tactile and non-contact sensors. This design is a promising strategy for a wide range of applications, including bionic e-skin, wearable electronics, and applications in intelligent robots.

## Data Availability

The data supporting the findings of this study are available from the corresponding author upon reasonable request.

## Supplementary Materials

The following are available online at https://www.mdpi.com/article/10.3390/mi13030404/s1, Figure S1: Schematic of embossing process, Figure S2: Hysteretic behavior, Figure S3: Non-contact sensing testing by subjects with different conductivity and polarity, Figure S4: Wireless sensing arrays in parallel reading principle.

## Appendix A

**Table A1:** Material parameters of PDMS.

Parameter	Description	Value
*E*	Young’s modulus	850 kPa
ν	Poisson’s ratio	0.49
ε_r_	Relative permittivity	2.75 (pure)

**Table A2:** Material parameters of Ecoflex.

Parameter	Description	Value
*E*_1_	Young’s modulus	600 kPa
ν_1_	Poisson’s ratio	0.49
ε_r1_	Relative permittivity	2.3

**Table A3:** Comparison of results.

Type	Numbers of Layers	Arrangement	Initial Frequency (MHz)	Sensitivity (MHz/kPa)	
Simulation	Single	6 × 6	290.4	–2.2	
		5 × 5	295	–3.2	
		4 × 4	297	–5.2	
Experiment	Double	5 × 5	276	Low-pressure	–14.25
				Mid-pressure	–2.86
				High-pressure	–0.60
