# Supplementary material for: Radio Frequency Resonator-Based Flexible Wireless Pressure Sensor with MWCNT-PDMS Bilayer Microstructure
Source: Micromachines (Basel). 2022 Mar 1;13(3):404. doi: 10.3390/mi13030404 (PMC8952374; doi:10.3390/mi13030404)
Supplement: Supplementary file 1 [file micromachines-13-00404-s001.zip › micromachines-1582563-supplementary.pdf]

Supplementary information

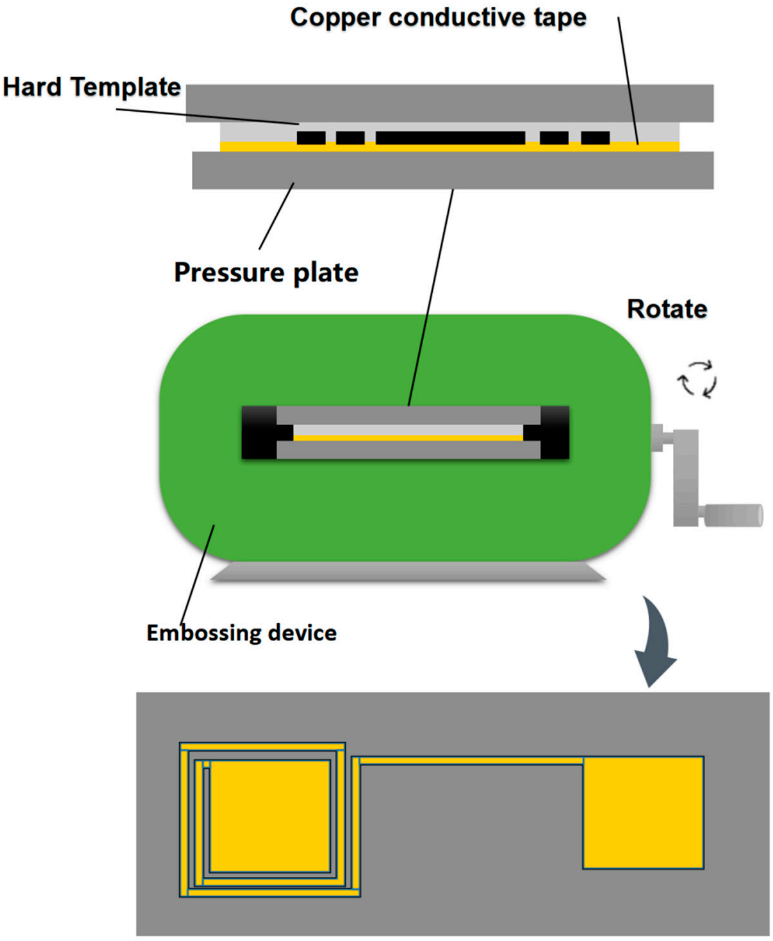

Figure S1. Schematic of embossing process.

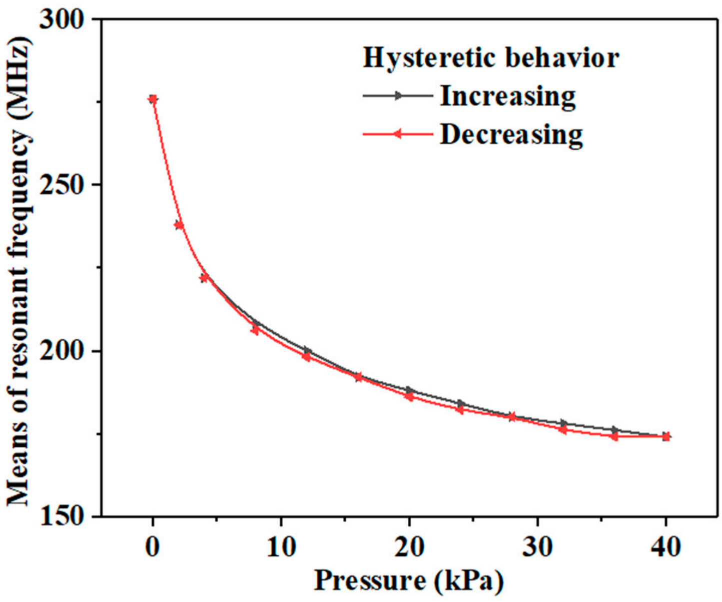

Figure S2. Hysteretic behavior.

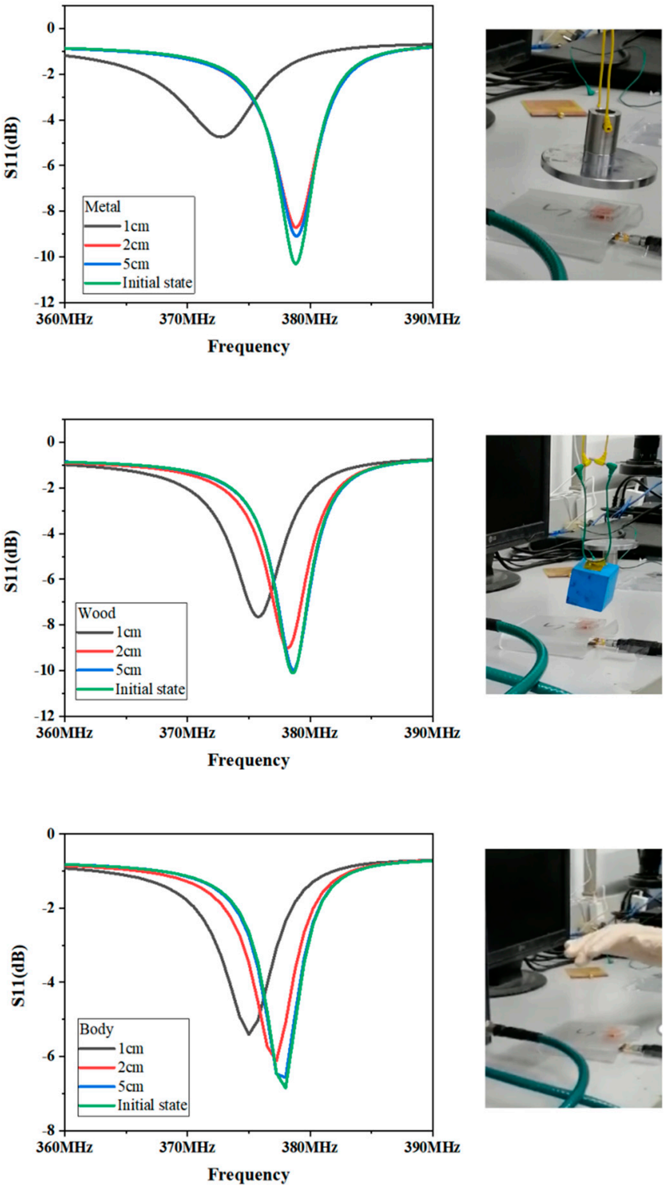

Figure S3. Non-contact sensing testing by subjects with different conductivity and polarity. The non-contact experiment compared three objects with different conductivity and polarity: metal, wood, and hand. From the comparison of the three data graphs in Figure S3, it can be seen that metal has the most obvious effect on the amplitude, while human body and wood have almost the same effect on the amplitude. This may be due to differences in electrical conductivity. The effect of metal, body, and wood on the resonant frequency is gradually weakened, but the difference is inconspicuous.

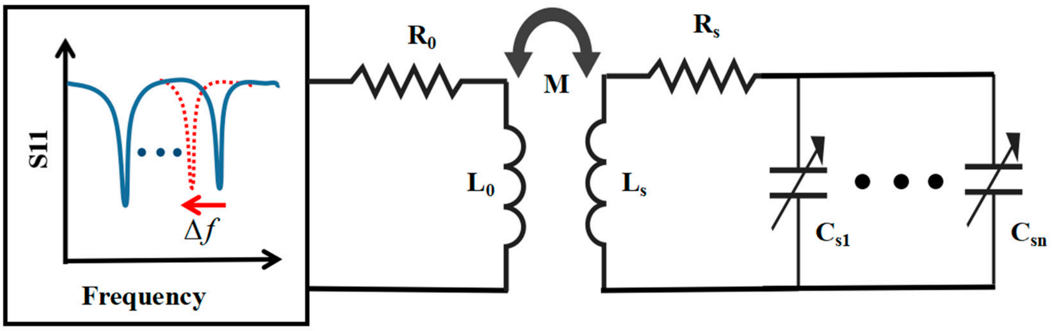

Figure S4. Wireless sensing arrays in parallel reading principle.
